# Impact of brief telehealth interventions on parental stress and challenging behaviors of children with fragile X syndrome

**DOI:** 10.1186/s13023-025-04019-1

**Published:** 2025-09-24

**Authors:** María Francisca Miranda, Víctor Faundes, María Angélica Alliende, Lorena Santa María

**Affiliations:** Human Genomics and Cytogenetics Laboratory, INTA, University of Chile, Av. El Líbano 5524, Macul, 7830490 Santiago de Chile, Chile

**Keywords:** Fragile X syndrome (FXS), Parent-implemented interventions (PII), Telehealth, Parental stress, Behavior

## Abstract

**Background:**

Fragile X syndrome (FXS) is the most common cause of inherited intellectual disability (ID) with comorbid autism and several support requirements. Challenging behaviors are frequently reported as a main concern for parents and caregivers, who also experience increased stress levels. There is little evidence of telehealth parent-implemented intervention (PII) for this population. Our study focused on describing the impact that brief telehealth parent-implemented interventions had on the parental stress levels and challenging behaviors of children with FXS in a Latin American country.

**Methods:**

Thirteen caregivers were assessed pre- and postintervention with the Parenting Stress Index short form (PSI-SF), Motivation Assessment Scale (MAS), and Fragile-X-specific adaptation of the Aberrant Behavior Checklist-Community questionnaire (ABC-C_FX_). Four telehealth sessions were developed with each participant to guide their intervention with their children with FXS. Statistical analysis was performed using paired t tests or Wilcoxon matched-pairs tests, and Pearson’s and Spearman’s correlations were used for comparisons. All the statistical analyses were performed using GraphPad Prism v8.3.0, and a two-tailed p value < 0.05 was considered to indicate statistical significance.

**Results:**

PSI-SF (TS_initial_=85(52.5–97) vs. TS_final_=55(27.5–90), *p* = 0.0117) and two MAS subscale frequencies of occurrence (scape_initial_=10(4-12.5) vs. scape_final_=3(0.5–8.5), *p* = 0.0146; tangible_initial_ =11.69 ± 8.27 vs. tangible_final_=7.154 ± 6.56, *p* = 0.0146) significantly decreased. ABC-C_FX_ did not significantly differ. The LSI-SF was positively correlated with three ABC-C_FX_ subindexes (lethargy/withdrawal s = 0.719, *p* = 0.007; hyperactivity *r* = 0.682, *p* = 0.01; and irritability s = 0.69, *p* = 0.011).

**Conclusions:**

Telehealth parent-implemented interventions decreased parental stress and challenging behavior perception and increased feelings of parental competence. The PII benefits interventions for children with FXS and is a key aspect to consider in situations where movement, transfer and access to specialized professionals are difficult or interfered with in a particular region or because of a major sanitary alert.

## Background

Fragile X syndrome (FXS) is a rare disease recognized as the most common cause of inherited intellectual disability (ID) and comorbid autism [1], with an estimated prevalence of 1:4,000 males and 1:8,000 females [2]. FXS is caused by an expansion of the trinucleotide CGG over 200 repeats, known as full mutation (FM), in the 5′ untranslated region of fragile X-messenger ribonucleoprotein gene 1 (*FMR1*), located at Xq27.3 [2]. This expansion causes hypermethylation of the *FMR1* promoter and consequently its silencing and lack of the fragile X-messenger ribonucleoprotein (FMRP). FMRP is a highly expressed protein that binds to multiple mRNAs to regulate their translation [3], which promotes synaptic plasticity and structural integrity in the brain [4].

The clinical manifestations of FXS include a variety of widely documented physical characteristics, behaviors and neurological impairments, such as anxiety, social withdrawal, attention deficit, impulsivity, developmental delays, speech and communication difficulties, irritability, self-injurious behavior, and hyperactivity [5, 6]. These impairments impact learning opportunities and daily functioning and generate social problems [5]. Furthermore, behavior problems are frequently reported in children with FXS, specifically in boys aged between 5 and 16 years, with a high prevalence of attention problems and distractibility, followed by overactive and impulse behavior [5]. Additionally, stereotypy and aggression are highly exhibited by males with FXS during adolescence [7], with the latter being the most prevalent behavioral problem [8, 9].

Challenging behaviors are commonly reported in individuals with FXS [9] and have been associated with increased stress levels in parents and main caregivers [10, 11]. Furthermore, the lack of emotional and psychological support for both children with FXS and their caregivers has a significant impact on stress management, well-being and parental functioning [12, 13]. Thus, stress among caregivers of individuals with disabilities has been associated with social isolation, lack of supportive networks and poor parenting confidence [13, 14].

Parent-implemented interventions (PIIs) involve parents in individualized intervention practices with their child, which increases positive learning opportunities, the acquisition of important skills and behavioral support by increasing parental skills and knowledge through didactic instruction, coaching/supervision and in-home practice assignments, among others [15–17]. PIIs can also increase parents’ engagement and feelings of competence [22,15, 18] and decrease parental stress and strain [19]. Recently, PIIs have been applied with parents of children with FXS focused on language improvement interventions [20, 21] and problematic behavior [22–24]. Additionally, PIIs show positive outcomes after intervention procedures, such as increased engagement time in activities, word number, and decreased problematic behavior, such as irritability, hyperactivity, and stereotypic behavior, among others, which remain in follow-up assessments [22, 23].

Despite the relevance of PIIs, little is known about the effect of telehealth-delivered PIIs on parenting stress, sense of competence and bonding in families with children with FXS. To our knowledge, there is only one study on the aforementioned subject, with promising results after a 12-week intervention period [22]. Telehealth was especially relevant during the recent COVID-19 pandemic and lockdown periods [25–28], but it has also been shown to be relevant in some situations where people cannot access the healthcare system in person or they live far from those centers (e.g., countryside) [27]. However, PIIs usually require a specific setting and conditions to be reproduced and/or standardized and thus to be delivered by telehealth to families with a child with disabilities, which could be difficult to reproduce or standardize in developing countries [22–24]. Despite these issues and considering that lockdowns increased overall mental problems and stress, especially in parents and people with disabilities [29, 30], we hypothesized that telehealth-delivered PIIs during the recent COVID-19 pandemic would be an effective tool for improving both parents’ satisfaction and problematic behaviors in families with children with FXS. Therefore, the aim of this study was to describe the impact of brief telehealth and parent-implemented interventions on the parental stress levels and challenging behaviors of children with FXS in a Latin American country.

## Methods

### Participants and study design

The participants were recruited through the Human Genomics and Cytogenetics Laboratory at INTA (MCL-INTA), University of Chile, in collaboration with the Fragile X Corporation of Chile, a national group of families with relatives with FXS.

Parents and/or main caregivers of a child with a confirmed molecular diagnosis of Fragile X Syndrome were considered to participate in the study. Additionally, only parents and/or main caregivers who did not participate in previous parenting interventions were invited to participate in the study. Families should have an electronic device with internet access and one of the following platforms: WhatsApp (video calls), Google Meet or Zoom. Once eligible families were identified, they were contacted to depict them the study and were asked to provide written informed consent for the research if they agreed to participate. The process was developed entirely through online telehealth interventions, and family groups from different Chilean regions that lack specialized professionals were prioritized. Once families were recruited, an interview was conducted to obtain sociodemographic and clinical data, and the families were asked to complete a set of psychometric tests (see Measurements subsection). These procedures allowed us to identify areas of concern/needs of individual families, child behaviors and their impact, parent‒child interactions, and support networks and/or resources.

Four telehealth sessions were delivered every two weeks. Personalized objectives were established for each family to provide support in three main areas: diagnosis awareness (psychoeducation), strategies of support for challenging behavior, and the development of functional activities. Each session was conducted following international recommendations for delivering PIIs. For this purpose, a certified clinical neuropsychologist taught, trained and collaborated with caregivers to implement evidence-based practices (EBPs) with their children through daily routines and activities [15].

**Table 1:** Description of parent-implemented interventions procedure

Session 1	Session 2	Session 3	Session 4
♣ Selecting goal ♣ Psychoeducation. ♣ Examples and behavior analysis. ♣ Strategies definition, use and explanation. ♣ Exchanges of ideas. ♣ Support document. ♣ Task.	♣ Monitoring task ♣ Review and behavior analysis ♣ Monitoring progress and barriers ♣ Psychoeducation ♣ Exchanges of ideas ♣ Task	♣ Monitoring task ♣ Review and behavior analysis ♣ Monitoring progress and barriers ♣ Psychoeducation ♣ Exchanges of ideas ♣ Task	♣ Monitoring task ♣ Monitoring progress and barriers ♣ Review and behavior analysis ♣ Psychoeducation ♣ Exchanges of ideas ♣ Critical analysis of the intervention process ♣ Closing Process

After completing the intervention process, parents were asked to complete the same psychometric tests used before this intervention and to provide feedback to improve the described intervention in the future.

### Measurements

The *Parenting Stress Index short form (PSI-SF)* was developed to assess stress levels in parents and has multiple clinical applications in several family contexts. The PSI-SF proposed a three-factor model: parenting distress (PD), a measure of anxiety due to personal factors related to parenting; parent‒child dysfunctional interaction (P-CDI), which focuses on parental perceptions of and expectations for their interactions with their children; and difficult child (DC), which assesses parents’ perceptions of child behavior and how difficult it can be to address such behavior [31, 32]. The total PSI-SF score is an indicator of the overall experience of parenting stress and considers parents’ features and feelings as well as children’s needs, adaptability, temper and bond [31, 33, 34]. *The* PSI-SF is the only translated and standardized instrument of its kind for the Latin American population, and it has been used in validity and reliability studies in our country [31]. The test-retest reliability ranged from 0.68 to 0.85, and the internal reliability ranged from 0.80 to 0.91 (31; 33).

The *Motivation Assessment Scale (MAS)* is a rating scale designed to identify the motivation behind problematic behavior in individuals with developmental disabilities 12 [35, 36]. Potential motivational behaviors are categorized into four subscales: *attention*,* escape*,* tangibles*,* and sensory consequences* [37]. The Spanish version was used for the assessment, and 0.92–0.98 are test-retest reliability values reported by the original authors [38].

*The Fragile-X-specific adaptation of the Aberrant Behavior Checklist-Community questionnaire (ABC-C*_*FX*_*)*, which comprises six subscales: irritability, lethargy/withdrawal, stereotypy, hyperactivity, inappropriate speech and social avoidance, was used to evaluate maladaptive behaviors [39]. Higher scores on the ABC-C_FX_ suggest greater maladaptive behaviors. Test-retest reliability has not been studied for the FXS adaptation of the test. However, prior work has reported good-to-excellent stability of the ABC-C in patients with FXS [39].

### Data analysis

Summary descriptive statistics are shown as absolute and relative frequencies (n[%]) for categorical variables, whereas means and standard deviations (M ± SDs) or medians and interquartile ranges (m[IQRs]) are shown for continuous, parametric or nonparametric variables, respectively (Table 1).

To evaluate the impact of the telehealth intervention on families, pre- and postintervention scores obtained for every set and subset of the described measurements were compared using paired t tests or Wilcoxon matched-pairs tests. Postintervention scores from children with FXS were subjected to bivariate analyses to study their correlations with other postintervention psychometric data, either from themselves or from their parents, as independent variables. For this purpose, Pearson’s and Spearman’s correlations were calculated. All the statistical analyses were performed using GraphPad Prism v8.3.0, and a two-tailed p value < 0.05 was considered to indicate statistical significance.

## Results

### Recruitment process

Initially, twelve families met the inclusion criteria for the study. Fourteen parents were assessed and asked to provide written informed consent. Only one family declined to continue the intervention process once it was initiated because of the unexpected health problems of another family member. Measurements of this family were not considered in the final data analysis; therefore, thirteen caregivers were ultimately considered for full interventions, and their data were analyzed.

### Demographic and clinical characteristics

The descriptive statistics of these features are presented in Table 2. Regarding sociodemographic data, caregivers were predominantly females (76.9%; *n* = 10), all of whom were mothers of individuals with FXS and all of whom were males. The mean age of the caregivers was 44 ± 8 years, while the mean age of the children with FXS was 12.72 ± 5,04 years. Ten out of 13 caregivers (76.9%) had a technical or university degree, and 46.2% (*n* = 6) had a full-time job. All of the caregivers identified their family as their supportive network, whereas the child’s educational establishment (61.5%; *n* = 8) and the healthcare system (30.76%; *n* = 4) were identified as the supportive network for their affected children. Regarding the intervention, all caregivers required diagnosis awareness, whereas 76.9% (*n* = 10) of them identified challenging behaviors as the main area of concern. Only three caregivers (23%) (*n* = 3) required assistance with the development of functional activities.

**Table 2:** Socio demographic features

FINDING ^a^	FREQUENCY OR DISTRIBUTION
Sex of caregiver (Females, n[%]) (*n* = 13)	10 [76,92%]
Age of caregiver participant (M ± SD)	44 ± 8
Sex of child with FXS (Males, n[%]) (*n* = 11)	11 [100%]
Age of child with FXS (M ± SD)	12,72 ± 5,04
Age of child diagnosis (M ± SD)	5,27 ± 3,71
Caregiver’s educational level (*n* = 13[%])	
Left school (undergraduate) (Level 1)	0 [0]
High school certificate (12 years of education) (Level 2)	1 [7,69%]
Career Technical Education incomplete (Level 3)	1 [7,69%]
Career Technical Education complete (Level 4)	4 [30,76%]
Professional qualification without a degree (Level 5)	1 [7,69%]
University degree (Level 6)	6 [46,15%]
Postgraduate degree (Level 7)	0 [0]
Employment status of caregiver(*n* = 13[%])	
Unemployed	2 [15,38%]
Part-time worker	1 [7,69%]
Full-time worker	6 [46,15%]
Independent worker	3 [23,07%]
Housekeeper	1 [7,69%]
Caregiver and/or child has a network support (*n* = 13[%])	13 [100%]
Caregiver has a physical or mental health disease (*n* = 13[%])	7 [53,84%]
Estimation of monthly expenses related to their child genetic diagnosis (M ± SD)	233 USD ± 112
Intervention focus (*n* = 13[%])	
Diagnosis awareness (Psychoeducation)	13 [100%]
Challenging behavior support strategies	10 [76,92%]
Functional activities development	3 [23,07%]

Note. Abbreviations: M = Mean; SD = Standard deviation

^a^ The number of responders are detailed for every feature when necessary, and their frequencies/distributions were calculated according to that number.

### Intervention measurements

Table 3 shows a comparative analysis performed for each psychometric variable assessed before and after the intervention process. While global parental stress levels decreased significantly after the intervention (PSI-SF: initial TS = 85 [52.5–97] vs. final TS = 55 [27.5–90], *p* = 0.0117), the parenting distress did not (PD: initial = 68.69 ± 28.11 vs. final = 57 ± 31.18, *p* = 0.0583). Both P-CDI and DC scores decreased after the intervention, but not significantly. Within the motivation assessment scale, the occurrence of escape behaviors and the ability to obtain a tangible object significantly decreased after the intervention (scape initial score = 10 [4-12.5] vs. scape final score = 3 [0.5–8.5], *p* = 0.0132; tangible initial score = 11.69 ± 8.27 vs. tangible final score = 7.154 ± 6.56, *p* = 0.0146). ABC-C_FX_ measurements did not reveal any significant difference after the intervention.

**Table 3:** Comparison of pre- and postintervention score measurements

	Initial score	Final score	*p* value^a^
Parental Stress Index short form (PSI-SF)			
Parenting Distress (PD) [M ± SD]	68,69 ± 28,11	57 ± 31,18	0.0583
P-CDI [m(IQR)]	80 (42.5–92.5)	65 (42.5–85)	0.2354
CDI [m(IQR)]	60 (25–95)	50 (15-67.5)	0.1982
Total score [m(IQR)]	85 (52,5–97)	55 (27,5–90)	**0.0117**
Motivation Assessment Scale (MAS)			
Sensory consequences [m(IQR)]	10 (3.5–13.5)	4 (1.5–15.5)	0.1309
Escape [m(IQR)]	10 (4-12.5)	3 (0.5–8.5)	**0.0132**
Attention [m(IQR)]	7(0.5–11)	6 (0.5-11-5)	0.9160
Tangibles [M ± SD]	11.69 ± 8.27	7,154 ± 6.56	**0.0146**
Aberrant Behavior Checklist-Community questionnaire (ABC-C_FX_)^b^			
Irritability [m(IQR)]	8 (3.5–22.5)	10 (4.5–15)	0.9834
Lethargy/Withdrawal [m(IQR)]	6 (2–13)	5 (1–9)	0.4707
Stereotypy [m(IQR)]	4 (1.5–7)	4 (1-8.5)	0.6953
Hyperactivity [M ± SD]	7.84 ± 5.98	7.69 ± 6	0.8078
Inappropriate speech [M ± SD]	4.46 ± 3.86	4.30 ± 3.03	0.8506
Social avoidance [M ± SD]	2.92 ± 1.70	3.07 ± 3.01	0.8243

*Note. Abbreviations*: M = mean; SD = standard deviation; m = median; IQR = interquartile range

^a^ Significant p values (*p* < 0.05) are depicted in bold.

^b^ The Fragile-X-specific adaptation of the Aberrant Behavior Checklist-Community questionnaire (ABC-C_FX_)

Table 4 provides data from bivariate analysis conducted to identify significant correlations between final scores of children’s psychometric data and other postintervention psychometric variables. The overall PSI-SF score was significantly and positively correlated with the irritability (s = 0.69, *p* = 0.011), lethargy/withdrawal behaviors (s = 0.719, *p* = 0.007), stereotypy (*r* = 0.632, *p* = 0.021), and hyperactivity (*r* = 0.682, *p* = 0.01) subindexes of the ABC-C_FX_ scale. No specific PSI-SF subscale showed significant correlations. Positive correlations were revealed between different subindexes of the ABC-C_FX_ and MAS scales. Hyperactivity (ABC-C_FX_) was positively correlated with three subscales of the MAS: sensory consequences (s = 0.631, *p* = 0.023), attention (s = 0.675, *p* = 0.014) and obtaining tangible objects (*r* = 0.668, *p* = 0.013), whereas inappropriate speech and social avoidance (ABC-C_FX_) were positively correlated with escape (s = 0.659, *p* = 0.017) and sensory consequences (s = 0.591, *p* = 0.037), respectively.

**Table 4:** Significant associations between PSI-SF subscales of MAS subscales and ABC-CFX subscalesa

Feature	Irritability		Lethargy/Withdrawal		Stereotypy		Hyperactivity		Inappropriate speech		Social Avoidance	
	Correlation^b^	*p* value	Correlation^b^	*p* value	Correlation^b^	*p* value	Correlation^b^	*p* value	Correlation^b^	*p* value	Correlation^b^	*p* value
PSI-SF												
PD	-	-	-	-	-	-	-	-	-	-	-	-
P-CDI	-	-	-	-	-	-	-	-	-	-	-	-
CDI	-	-	-	-	-	-	-	-	-	-	-	-
TS	s = 0.69	0.011	s = 0.719	0.007	*r* = 0.632	0.021	*r* = 0.682	0.01	-	-	-	-
MAS												
Sensory Consequences	-	-	-	-	-	-	s = 0.631	0.023	-	-	s = 0.591	0.037
Escape	-	-	-	-	-	-	-	-	s = 0.659	0.017	-	-
Attention	-	-	-	-	-	-	s = 0.675	0.014	-	-	-	-
Tangibles	-	-	-	-	-	-	*r* = 0.668	0.013	-	-	-	-

*Note. Abbreviations*: PSI-SF = Parenting Stress Index short form; PD = Parenting Distress: P-CDI = Parent‒Child Dysfunctional Interaction; CDI = Difficult Child; TS = Total score; MAS = Motivation Assessment Scale

^a^Non-significant (NS) associations were not depicted in this table for better visualization of the remaining results.

^b^Spearman orPearson’s correlations were conducted according to parametric or nonparametric distribution of variables

## Discussion

This study describes for the first time the impact of brief telehealth-delivered parent-implemented interventions on the parental stress levels and challenging behaviors of children with FXS in a Latin American country. Telehealth interventions geared toward parents significantly decreased overall perceived stress levels (PSI-SF TS scores) and parenting distress (PD scores) and decreased motivation behind target problem behaviors in children with FXS (Table 3).

Mothers of children with FXS were the main subject of this study (Table 2), corresponding to predominant family-caregiving roles associated with females as primary caregivers [40, 41]. Because of the gender gap, women are more likely to provide more personal and instrumental support [42–44] and spend more time on caregiving than men [45], with consequences for their physical and mental health and social involvement [40, 41, 44–46]. In general, stress related to caregiving has immediate negative effects, which are related to depression, poor health and low self-efficacy [42]. One particular study showed that loneliness in the caregiver population was more prevalent than depression and could serve as an early indicator that caregiver-related stress may be out of balance with caregivers’ personal resources [47]. In contrast, protective factors for caregivers to reduce stress levels included self-efficacy, support networks and active coping strategies [47].

Previous research has shown that parents of children with FXS experience greater stress than do parents of typically developing children, and poor parenting competence has been found to be one of the major stress determinants [12, 14]. Our results revealed that decreasing stress levels after a brief intervention period were specifically related to (1) overall perceived stress (Table 3: PSI-SF TS) and (2) parental distress (PD) related to parents’ features and their own perceptions of their role [34]. Research has also shown significant negative correlations between parental stress, social support and satisfaction support [13, 22]. Furthermore, social support plays a significant role in relieving stress, which is linked to individuals’ subjective perception that they will receive the desired support and help in a timely way from their surrounding social networks [48, 49].

Interestingly, although challenging behaviors did not significantly decrease from the parents’ perspective (Table 3), positive correlations were found between reported challenging behaviors, parental stress levels and the motivation behind specific behaviors (Table 4). Potter et al. [50] suggested that the perception of parents about their child’s behavior contributes more to their stress than their adjustment to parenting or their relationship with their child. Therefore, correlations between the overall PSI-SF score and lethargy/withdrawal behaviors, hyperactivity, and irritability could be related to increased awareness of FXS—specifically about neuropsychological features—and to the successful development of effective strategies designed to diminish children’s challenging behaviors, which in turn improves parental competence [13, 22, 51]. Similarly, correlations between the ABC-C_FX_ and MAS subscales (Table 4), specifically hyperactivity and inappropriate speech, could be considered indicators of the abovementioned parent-mediated intervention benefits because of a better understanding of motivations behind a specific behavior, functional communication and appropriate use of resources.

This study emerged during the pandemic lockdown as an opportunity to continue providing specialized therapeutic interventions to individuals with FXS and their families, particularly to those unable to access specialized medical care centers. Therefore, this study was planned to address a clinical need. However and due to the limited evidence on the topic [17, 22], this work was then developed to add scientific evidence on the impact of telehealth-based interventions, following recommendations for its design and measuring objectively its effectiveness. In other words, our intervention helped to deliver a clinical management for individuals with multiple developmental impairments, but using scientific, academic methods to demonstrate its effectiveness. However, in the future it would be useful to compare how the results of the intervention change in a non-academic setting.

While PIIs have been widely recognized for the treatment of autism spectrum disorders in face-to-face settings [16, 17], there is a notable scarcity of studies specifically addressing such approaches in the context of Fragile X Syndrome [17, 20]. Therefore, in the future it will be relevant to adapt this intervention to a face-to-face setting with a larger sample in order to see if these results are replicated. This step is crucial to demonstrate the effectiveness of this intervention independently of the way of delivery.

Despite the interesting results of our study, some limitations must be considered in its analysis in terms of sample size and intervention period. Follow-up assessment after a specific time period should be considered in future studies. Moreover, interactions between parents and children with FXS could be an interesting aspect to explore in future research, and more interventions designed to increase the knowledge and understanding of the impact of telehealth and parent-implemented interventions on parental stress levels and challenging behaviors of children with FXS should be planned to recommend these tools with strong evidence.

## Conclusions

Parent-implemented interventions through telehealth after a brief time period proved to be effective in diminishing parental stress levels and increasing parents’ understanding of challenging behaviors and motivation behind target behaviors in children with FXS, which enhanced parenting competence. Access to specialized professional support networks, including parental coaching, benefits children’s interventions and must be considered a significant aspect to diminish loneliness, feelings of isolation, and parental stress levels, especially in situations where moving, transferring, and accessing specialized professionals are difficult in a particular region or with a major sanitary alert.

## Data Availability

The datasets used and/or analyzed during the current study are available from the corresponding author upon reasonable request.

## Abbreviations

FXS: Fragile X syndrome
ID: Intellectual disability
FMR1: Fragile X messenger ribonucleoprotein gene 1
FMRP: Fragile X messenger ribonucleoprotein (FMRP)
PII: Parent-implemented interventions
PSI-SF: Parental stress index short form
MAS: Motivation assessment scale
ABC-CFX: Fragile-X-specific adaptation of the Aberrant behavior checklist-community questionnaire
PD: Parenting distress
P-CDI: Parent‒child dysfunctional interaction
DC: Difficult child
